# Medical Opinion and Movement

**Published:** 1910-11-05

**Authors:** 


### Medical Opinion and Movement.

### Urticaria from the Sun's Rays.

A curious and unusual case is recorded by Dr. B. F. Ochs in the *Medical Record* of an urticaria caused by exposure to the sun's rays. The patient was a woman aged thirty-four, who enjoyed excellent health and had no troubles either of digestion or menstruation. For nine years she had observed that whenever any part of her body was exposed to sunlight—arm, leg, or face—an urticarial eruption developed in that part, the intensity of which was in direct proportion to the quantity of light. If the day was dull the eruption would be slight, but on a sunny day it would be very marked. The author had seen the patient on several occasions when there was no rash, and it was sufficient then to uncover the arms and expose them to the sun to see appear at the end of a few moments characteristic patches of urticaria. So long as the patient provided herself with an umbrella of dark shade and black gloves there would be no eruption. In order to determine that the urticaria was really due exclusively to exposure to the sun, Dr. Ochs instructed the patient to consume all manner of food likely to set up such an eruption, at the same time keeping herself protected from the sun; but no rash appeared until she was again exposed to the sun's action.

### Stroganov's Treatment of Eclampsia.

Some years ago Professor Stroganov, of St. Petersburg, advocated the treatment of puerperal eclampsia by means of morphia and chloral instead of by the administration of chloroform, his chief object being to avoid the pulmonary complications and the aggravation of the toxæmia by the action of the anæsthetic on the lungs and liver. Hitherto the method has not received much favour outside Russia, and the few attempts that have been made to follow his line of treatment have not been very successful. Comparisons, however, between mortality statistics afford overwhelming evidence of the efficacy of Stroganov's methods. According to his latest report, he has treated 360 cases of eclampsia with a mortality of 6 per cent. Against this the mortality at Berlin is 14 per cent.; at Leipzig, 15 per cent.; in Mecklenburg-Schwerin, 24.4 per cent.; in France, 20 to 25 per cent.; and in the City of New York Lying-in Hospital, 30.8 per cent. On the other hand, Dr. E. Roth has recently given the method an extended trial at Dresden in a series of thirty-one cases, and is able to record only one death. The treatment consists in the administration of morphia and chloral in repeated doses so as to prevent the attacks of convulsions. Nothing further is done except that the patient is kept absolutely quiet in silence and darkness. Any attention to the patient—hypodermic injection, douching, vaginal examination, or catheterism—is carried out under light chloroform anæsthesia. At the onset of labour a hypodermic injection of 15 milligrammes of morphia hydrochloride is given, followed an hour later by a rectal injection containing 2 grammes of chloral hydrate. At the third hour a second morphia injection is given, at the seventh a second dose of chloral hydrate, and at the thirteenth and twenty-first hours further doses of 1.5 gramme chloral hydrate. On the second day, if the child is delivered and the patient is progressing, three more doses of 1.5 gramme of chloral hydrate are administered. If, however, the convulsions persist, rapid delivery is effected or Cæsarean section is performed.

### A New Sign of Paralysis.

Drs. Pierre Marie and Foix draw attention to a new sign observed by them in certain cases of paralysis of organic origin. It is produced by a slow and progressive, but forced, flexion of the toes, avoiding at the same time a forced extension of the foot. When this is carried out in a patient affected with paralysis it excites a reflex movement of the lower limb, consisting of flexion of all three segments of the limb, together with internal rotation and adduction of the foot. This movement is slow and regular, and the limb remains in the final position for a certain length of time after cessation of flexion of the toes. Transverse pressure of the foot produces the same result, but it may only induce a contraction of the leg. The authors have observed this sign in almost all cases of spastic paralysis, of hemiplegia, and of the spastic familial diseases. In hemiplegia it is as constant and early a symptom as the sign of Babinski. The reflex movement is closely analogous in its form to that described by Claude in hemiplegics; but the authors have not been able to attach to it the same prognostic significance.

### Cystoscopy of the Serous Cavities.

Dr. H. C. Jacobeus has conceived the idea of making a direct inspection of the peritoneal and pleural cavities by means of a cystoscope. He uses a trocar and cannula, and a cystoscope of Nitze which is sufficiently small to pass within it. He first makes a small incision in the skin and passes in the trocar, empties the cavity of fluid, and insufflates air by means of a Politzer air-pump. He then introduces the cystoscope, which is fitted with a valve to prevent the air escaping. The manœuvre is easier in the case of the pleura than of the peritoneum, on account of the danger of injury to the viscera, but experiments on the cadaver have shown that the introduction of the trocar does not involve the risk of injury to the intestines, even when there is no ascites. The author has been able by this means to observe the presence of hepatic cancerous nodules, of peri-hepatitis, peritoneal congestion, cancerous nodules of the intestine, and to study intestinal peristalsis. In the case of the pleura he has not been able to detect definite lesions, but hopes to succeed in doing so with more dexterity. He is of opinion that " laparoscopy " and " thoracoscopy " should furnish useful data, comparable in some degree with those of exploratory laparotomy. Considering, however, the somewhat complicated and difficult technique and manipulation, and the very limited range of observation obtainable even in the most favourable circumstances, it may be doubted whether the method offers any but a very limited field of usefulness.

### Nasal Cauterisation for Asthma.

The treatment of asthma by applying the actual cautery to certain spots on the interior of the nose is now a recognised practice which, though not infallible, does definitely relieve many sufferers from that intractable neurosis. Dr. Alexander Francis, who popularised it some years ago, first in Australia and then in England, read a paper on the subject before the British Medical Association which is now published in the *Journal of Laryngology*. What is the *rationale* of this remarkable effect? He thinks that suggestion plays no part in it in most cases, because many of the best results are obtained in the most incredulous patients; good results are also common among children too young to know the object of the treatment. Again in some cases cure has been effected after prolonged treatment in patients who had given up all hope; and the lightest touch of the cautery is often more successful than a severe application. The prognosis is, he says, better when the nose appears normal and when there are no signs of polypus or polypoid degeneration. Hence he excludes the irritative theory of relief. Since adopting Dr. Hare's view that asthma is a vasomotor neurosis he has found it possible to relieve other diseases by the same treatment, such as angina, migraine, Raynaud's disease, and mucous colitis. He can also reduce high blood-pressure by some 20 mm. of mercury in the same way; although there is a tendency to a subsequent rise, by persevering a permanent reduction of 20 to 40 mm. can be obtained. Dr. Francis believes that these vasomotor changes are due to action through the sympathetics of the cutaneous vessels, a not very lucid explanation of the reflexes concerned. He sounds a note of warning against indiscriminate nasal cauterisation. Several subsequent speakers opposed Dr. Francis, both in theory and practice, but others agreed with him as to the beneficial results of the cauterisation treatment.

### The Lævulosuria Test.

According to Dr. Hamilton, writing in the *Montreal Medical Journal*, a useful test, which is both simple in application and trustworthy in practice, for gauging the functional activity of the liver is provided by his method, based on the conversion of lævulose. The liver has been shown by Sachs to be the only organ capable of converting lævulose (into glycogen). When the liver is healthy no traces of lævulose can be found in the urine after the ingestion of three ounces of lævulose, because the whole is converted by the liver; but if the liver is diseased, lævulose can readily be detected by urinalysis. The patient, after a night's fasting, empties his bladder and is then given the above quantity of lævulose in water or weak tea, and four specimens of urine are examined at intervals of an hour. In cases of cirrhosis ninety-three cases were positive, according to this test, out of a hundred and two; seven out of nine of catarrhal jaundice; two out of three of jaundice with gall-stones; eleven out of sixteen of hepatic syphilis; and eight out of eight of obstruction of the common bile-duct. On the other hand, in passive venous congestion there were twenty-two negative reactions out of twenty-three.

### Frequency of Perineal Tears in Labour.

The custom which prevails almost universally in Great Britain of conducting the expulsive stages of labour in the lateral position is one which constantly excites unfavourable comment among Continental, Irish, and some American obstetricians. The objectors frequently allege difficulties in maintaining an aseptic technique in the lateral posture, and many advantages are claimed for the dorsal method. It is interesting to note that in the United States there is less unanimity on this subject than in Europe, and that quite a fair minority adopt the English fashion. Dr. W. B. Doherty, in *International Clinics*, makes a strong point of the frequency of ruptured perineum in the dorsal position. He states that 65 per cent. of women thus delivered sustain this accident, whereas in the lateral posture the proportion is no more than 25 per cent. He admits that more care and attention are required with the latter method, but says its wisdom cannot be questioned. In the same article he is emphatic on the necessity for rest in bed in the treatment of troublesome vomiting during pregnancy. A thorough preliminary purgation, with light diet, complete rest, and a physician who encourages the patient strongly to believe in cure: these will check hyperemesis in the vast majority of cases. He adds a word of warning about retroversion; the practitioner in dealing with any case of vomiting in early pregnancy should always be on the lookout for this contingency, and should make sure that it does not exist before proceeding to treatment.

### Acidosis in Children.

In the *British Journal of Children's Diseases* Dr. J. G. Sharp describes certain cases of idiopathic acidosis in children—that is to say, of acidosis the origin of which remains obscure. The symptoms are not unlike those of acidosis after chloroform inhalation, and there is the same characteristic sweet odour in the breath as in terminal cases of diabetes. The patient is drowsy, or even semicomatose, but rouses up when vomiting comes on. This is excited whenever any food is taken. There is loss of appetite, and intense thirst. Headache is frequently felt, but not very severely. The skin is hot and dry, and respiration is frequent. The temperature ranges from 99° to 105° F. The bowels are constipated and the urine scanty; the latter always contains acetone, and often diacetic acid, but not sugar. In mild cases the urgent symptoms last no longer than one or two days; a free action of the bowels is a good prognostic sign. In the severest cases, which end fatally, convulsions, cold sweats, and Cheyne-Stokes breathing are events which foreshadow the end. There is never any abdominal pain, peristalsis, or swelling; sometimes there is tenderness in the upper abdomen. The differential diagnosis has to be made chiefly from meningitis, which is much more gradual in onset, and is associated with irritability in the early stages; and from a " bilious attack " by the drowsiness and absence of headache. Several illustrative cases are reported, including two which ended fatally.

### Narcotics Preliminary to Anæsthesia.

In a paper contributed to the *Medical Record* Dr. Gwathmey emphasises certain aids to successful anæsthesia whose use he has found highly beneficial. Thus he is a strong advocate of a preliminary injection of morphia with atropine, no matter what drug is to be used for induction of anæsthesia. He lays down four canons: (1) When morphine is given, it should always be before, not after, the operation, in order to obtain the benefits of the drug in the induction and maintenance of anæsthesia; (2) all athletes and alcoholics should have this preliminary dose of morphine; (3) in the very old and the very young, if morphine is given at all, it should be administered with great caution; (4) whenever morphine is employed a lighter narcosis should be maintained than when it is not. He also insists strongly upon the value of warmed chloroform, which is much less dangerous than cold; he urges that a temperature of 100° F. should be maintained in the drop bottle, and that a water-bath should be used to restore this temperature as its contents get cool. With the idea of promoting rapid recovery and minimising nausea and vomiting, he applies hot towels to the patient's face as soon as the anæsthetic is discontinued. The author is very scornful about the prejudices of American surgeons, who oppose in many cases the appointment of skilled special anæsthetists in hospitals, and entrust the anæsthetic to a nurse. The result is that they have to employ open ether as a routine measure because of its comparative safety; selection of the anæsthetic to suit the patient is thus impossible. He states that nitrous-oxide gas and oxygen can be satisfactorily used for 30 per cent. of surgical cases, and that patients should have the benefit of this non-poisonous combination whenever possible. It is also noteworthy that Neu, in the *Münchener Medizinische Wochenschrift* regards the ideal anæsthesia as that produced by combining morphine, scopolamine, nitrous oxide, and oxygen.

### Quinine and Ectopic Gestation.

Although the published text-books are distinctly shy of dealing with the effects of quinine upon the reproductive functions, there is no doubt that there is an important relation between the two. Quinine has probably a slight ecbolic function, much more marked in some women than in others. It is well known, too, that it is a powerful poison to spermatozoa, both locally in the seminal fluid and when absorbed into the blood-stream. There are admittedly many men who live in the tropics, take quinine regularly, and yet preserve their procreative powers unabated; but of the majority it is probable that regular quinine-eating renders them infertile, relatively or actually. The same is believed to hold good of women, though it is naturally difficult to be quite sure how far these effects are due to quinine and how far to the malarial infections so common in the tropical regions where quinine prophylaxis is most indulged. In the *Medical Record* an American physician, Dr. Lemchen, puts forward the hypothesis that ectopic gestation may be favoured by the abuse of quinine. He argues that quinine is a protoplasmic poison with a specific action on cells having amœboid movements, and may thus interfere with the physiological action of the ciliated epithelium of the Fallopian tubes. Thus these cells fail to pass the impregnated ovum along the lumen of the tube into the uterus, and it then implants itself in one or other of the situations wherein it is subsequently found as an ectopic pregnancy. This hypothesis may possibly help to explain the fact that the latter condition is certainly commoner in the United States than in Great Britain; but it will be necessary to prove that it is commoner in the Southern States, where malaria is endemic, than in the North and in Canada. As a speculation it is distinctly of interest.

### Bleeding after Paracentesis Abdominis.

In view of the large number of cases in which paracentesis abdominis is performed for the removal of accumulations of ascitic fluid, it is perhaps a matter for wonder that injury of the superficial epigastric artery is a relatively infrequent accident. It is the practice of the French school to perform these operations in the middle line, not as is usually the practice, in the flank, to avoid injuring this vessel, and that their method is the safer one would seem to be demonstrated by a case reported in *La Tribune Médicale* by Foy and Woimant. The subject of their report was a woman aged forty-eight suffering from alcoholic cirrhosis of the liver associated with ascites and œdema of the legs. To relieve dyspnœa and distension, paracentesis was performed midway between the umbilicus and the left anterior superior spine. During the next half-hour 11 litres of bile-stained fluid were evacuated. The patient was then turned on her side, the cannula became slightly displaced, and a few drops of bright blood escaped. The possibility of there being some injury to the epigastric artery caused a careful watch to be placed on the patient, with the result that in half-an-hour definite symptoms of internal bleeding became manifest. An incision was then made slightly below the puncture left by the trochar. A fairly large cavity filled with clots was found between the muscles and the peritoneum. On removing the clots the divided ends of the vessel came into view, and it was an easy matter to secure these by ligatures. The wound was closed, after providing for free drainage, and the patient made a rapid recovery.

